# Novel susceptibility loci identified in a genome-wide association study of type 2 diabetes complications in population of Latvia

**DOI:** 10.1186/s12920-020-00860-4

**Published:** 2021-01-11

**Authors:** Monta Ustinova, Raitis Peculis, Raimonds Rescenko, Vita Rovite, Linda Zaharenko, Ilze Elbere, Laila Silamikele, Ilze Konrade, Jelizaveta Sokolovska, Valdis Pirags, Janis Klovins

**Affiliations:** 1 Latvian Biomedical Research and Study Centre, Ratsupites iela 1, Riga, 1067 Latvia; 2 Faculty of Medicine, University of Latvia, Jelgavas iela 3, Riga, 1004 Latvia; 3 Faculty of Medicine, Riga Stradins University, Dzirciema iela 16, Riga, 1007 Latvia

**Keywords:** Type 2 diabetes mellitus, Genome-wide genotyping, Diabetic complications

## Abstract

**Background:**

Type 2 diabetes complications cause a serious emotional and economical burden to patients and healthcare systems globally. Management of both acute and chronic complications of diabetes, which dramatically impair the quality of patients' life, is still an unsolved issue in diabetes care, suggesting a need for early identification of individuals with high risk for developing diabetes complications.

**Methods:**

We performed a genome-wide association study in 601 type 2 diabetes patients after stratifying them according to the presence or absence of four types of diabetes complications: diabetic neuropathy, diabetic nephropathy, macrovascular complications, and ophthalmic complications.

**Results:**

The analysis revealed ten novel associations showing genome-wide significance, including rs1132787 (*GYPA,* OR = 2.71; 95% CI = 2.02–3.64) and diabetic neuropathy, rs2477088 (*PDE4DIP*, OR = 2.50; 95% CI = 1.87–3.34), rs4852954 (*NAT8*, OR = 2.27; 95% CI = 2.71–3.01), rs6032 (*F5*, OR = 2.12; 95% CI = 1.63–2.77), rs6935464 (*RPS6KA2*, OR = 2.25; 95% CI = 6.69–3.01) and macrovascular complications, rs3095447 (*CCDC146*, OR = 2.18; 95% CI = 1.66–2.87) and ophthalmic complications. By applying the targeted approach of previously reported susceptibility loci we managed to replicate three associations: *MAPK14* (rs3761980, rs80028505) and diabetic neuropathy, *APOL1* (rs136161) and diabetic nephropathy.

**Conclusions:**

Together these results provide further evidence for the implication of genetic factors in the development of type 2 diabetes complications and highlight several potential key loci, able to modify the risk of developing these conditions. Moreover, the candidate variant approach proves a strong and consistent effect for multiple variants across different populations.

## Background

The past few decades have shown a marked increase in the number of patients with diabetes rising from 151 million (4.6% of the global population) in 2000 to 463 million (9.3%) in 2019 [1]. The risk of type 2 diabetes (T2DM), the most common type of diabetes, is modified by a strong interaction between environmental and genetic factors [2, 3]. T2DM is a multifactorial disease with a population-specific heritability (26% in the European population) [4]. A number of common variants implicated in the pathogenesis and genetic architecture of T2DM have been identified so far, some of them also capable of modifying the pharmacologic response to antidiabetic drugs [5, 6].

Uncontrolled T2DM can lead to long-term illnesses or chronic health conditions divided into microvascular complications, such as diabetic retinopathy, nephropathy, neuropathy, and macrovascular complications, including stroke, heart disease, and peripheral vascular disease [7], accounting for 53% of direct health costs of diabetes with cardiovascular and renal complications contributing to the greatest financial burden [8, 9]. Moreover, diabetic patients show increased all-cause mortality rates, especially cardiovascular deaths (HR 2.6, 95% CI 1.4–4.7) [10].

Studies show evidence of considerable genetic component predisposing to diabetic complications, explaining even around 50% of the risk of proliferative retinopathy [11]. In the last few decades, genetic research including genome-wide association studies (GWAS), linkage analysis, and candidate gene approach has revealed several susceptibility loci for diabetic retinopathy and nephropathy (*VEGF*, *CAT*, *FTO*, *UCP1,* and *INSR*), and also macrovascular complications (*ADIPOQ*). Nevertheless, they explain only a small proportion of the phenotypic variation observed in T2DM patients [12–17], justifying a need for identification of novel genetic risk factors for T2DM complications and improvement of knowledge about molecular mechanisms underlying these comorbid conditions.

Since the high impact of population specificity for the discrimination of genetic variants and their contribution to the phenotype of interest is evidenced by a number of SNPs that failed to replicate in different populations, both discovery and replication studies in populations of different ancestries are needed [18, 19]. An example of population-specific allele frequency is rs61736969, the risk variant of T2DM, which is located in the *TBC1D4* (TBC1 Domain Family Member 4) gene. It is highly frequent (minor allele frequency of 17%) in the Greenlandic population, nevertheless, it has not been present so far in European individuals, most probably due to different linkage disequilibrium patterns [20]. Although poor glycemic control is considered to be the driving factor of T2DM complications, early genotype-based identification of individuals at high risk of diabetic complications may promote the prevention or, at least, delay of the disease [21]. In this study, we aimed to discriminate novel susceptibility loci for T2DM complications and replicate the findings of other GWAS in the study cohort from the Genome Database of the Latvian Population (LGDB) [22].

## Methods

### Study group and phenotype definitions

In total, the study cohort consisted of 601 T2DM patients of European ancestry with and without a medical history of diabetes complications, selected from the participants of LGDB (recruited from June 2007 to November 2016) according to the following inclusion criteria: (1) clinically confirmed diagnosis of T2DM (E11 diagnosis code, ICD-10), (2) information on age at diagnosis, sex, weight, and height available, (3) records of national diabetes registry and Latvian hospital inpatient discharges available. Written broad consent was obtained from every subject during the recruitment in LGDB.

The collection of blood samples and relevant anthropometric data was ensured by LGDB according to their standard procedures [22]. Associated clinical data, including the diagnosis date of T2DM, date and type of diabetes complications, HbA1c measures, and medications used, were obtained from the records of Diabetes registry, Latvian hospital inpatient discharges, outpatient progress notes, and pharmacy recipe records, provided by The Centre for Disease Prevention and Control of Latvia and National Health Service of Latvia (Approval No. 3, Decision No. 7.1–3/3). The data about diabetic complications present for T2DM patients involved in LGDB were applied for accurate stratification of 601 T2DM patients in four phenotype-based groups according to the type of complications experienced: diabetic neuropathy, diabetic nephropathy, ophthalmic complications, and macrovascular complications. The definition of phenotypes and patient stratification in different complication groups was done as follows:

1. Diabetic neuropathies: clinical diagnosis codes (ICD-10) E11.4 and E11.5, records of amputation of the leg/toe, gangrene, shunting and angioplasty, and presence of intermittent claudication or fresh ulcers since the diagnosis of T2DM.
2. Diabetic nephropathies: clinical diagnosis code E11.2 or records of kidney failure, kidney transplantation, renal replacement therapy, microalbuminuria, hemodialysis, peritoneal dialysis performed after the diagnosis of T2DM.
3. Ophthalmic complications: clinical diagnosis code E11.3 or records of photocoagulation, maculopathy, retinopathy, operative therapy, blindness made since the diagnosis of T2DM.
4. Macrovascular complications: clinical diagnosis codes I95, I20, I21, I24, I25, I50, I60, I61, I63, I64, and records of coronary shunting and angioplasty after the diagnosis of T2DM.

ICD-10-based phenotype definitions corresponding to the Latvian guidelines of diabetes management are generally used in clinical practice in Latvia. Subjects with the above-mentioned diagnosis codes or medical events recorded were considered as cases in their corresponding complication groups, while T2DM patients with no evidence of complications of interest during their follow-up period were recognized as controls in the particular group. Subjects experiencing specific diabetes complications before the set of T2DM diagnosis were excluded from the analysis of a particular complication group, explaining the variable total number of individuals among all complication groups tested. The follow-up period which coincides with diabetes duration was considered as time since the set of T2DM diagnosis until the date of diabetes complication recorded for cases or the date of the last entry in the National registry for control subjects. Administration of medications was considered in a group-specific manner, accounting for angiotensin II receptor blockers and angiotensin-converting enzyme inhibitors in the analysis of all complication groups analyzed and additional lipid-modifying agents in the analysis of macrovascular complications. In order to adjust for the inter-individual variability of glycaemic control, a key factor in the development of T2DM complications, the median HbA1c level during the observation period was fitted as a covariate, irrespective of the antidiabetic therapy used. Sex, age at the diagnosis of T2DM, body mass index (BMI), diabetes duration, and use of particular medications were also included among the covariates.

### DNA extraction and genotyping

Within the framework of this study DNA samples from 601 T2DM patients were used. DNA was isolated from peripheral blood leukocytes using a phenol–chloroform extraction method according to LGDB standard procedures [22]. DNA samples were genotyped with the Infinium Global Screening Array (Illumina, USA) on the iScan System microarray scanner (Illumina, USA). Illumina Genome Studio v2.0 was used to convert raw data into PLINK format and workflow described in Marees et al. [23]. used for data quality control. SHAPEIT v2.r900 [24] and IMPUTE2 [25] were used for genotype phasing and genotype imputing. Imputed data were filtered using the following parameters: marker correlation (INFO) > 0.8, hard call threshold 0.1, minor allele frequency > 1%, Missingness < 2%.

### Statistical analysis

Association analyses corresponding to four different complication groups (macrovascular complications, diabetic neuropathy, diabetic nephropathy, ophthalmic complications) were performed using PLINK v1.9 logistic regression with covariates: median HbA1c, sex, age at the diagnosis, diabetes duration, BMI, medications used. A genome-wide significance threshold of *P* < 5 × 10^−8^ was defined.

For the targeted analysis candidate variants were selected from GWAS Central [26] (http://www.gwascentral.org/) and GWAS Catalog [27] (https://www.ebi.ac.uk/gwas/home), based on previously reported association with T2DM complications: diabetic neuropathy (4 allelic variants selected), macrovascular complications (43 allelic variants selected), ophthalmic complications (98 allelic variants selected) and diabetic nephropathy (49 allelic variants selected). A complete list of selected SNPs, their positions, and associated traits is provided in Additional file 1. False discovery rate (FDR) according to the Benjamini–Hochberg procedure was calculated to account for multiple testing and the threshold was set at 0.05.

Manhattan plots and Q-Q plots were generated in R v3.5.3 using the qqman package, while the Venn diagram was developed in the online visualization tool Venny 2.1.0. Statistical analysis of anthropometric measures and biochemical data was performed in R v3.5.3. by applying the Wilcoxon rank-sum test and Pearson's chi-squared test with a p-value threshold < 0.05. For identification of the functional role of allelic variants expression quantitative trait locus (eQTL) analysis was conducted by using the open-access Genotype Tissue Expression (GTEx) database [28]. The tissue types for eQTL analysis were carefully selected considering the etiology of the diseases (artery, nerve, heart, skin, blood) [29]. The p-value threshold of 0.05 was used to discriminate significant associations. Variant Effect Predictor and Linkage Disequilibrium (LD) data from 1000 Genome project (Utah Residents (CEPH) with Northern and Western European Ancestry) were employed to explore the functional consequences of each variant and other variants in LD [30]. To evaluate the potential SNP effects on quantitative phenotypes, analyses of variant association with HbA1C and BMI were performed using PLINK v1.9—assoc function for quantitative phenotypes, where the genome-wide significance threshold of *P* < 5 × 10^−8^ was used to identify the significant hits.

## Results

### Genome-wide association analysis

**Fig. 1:**
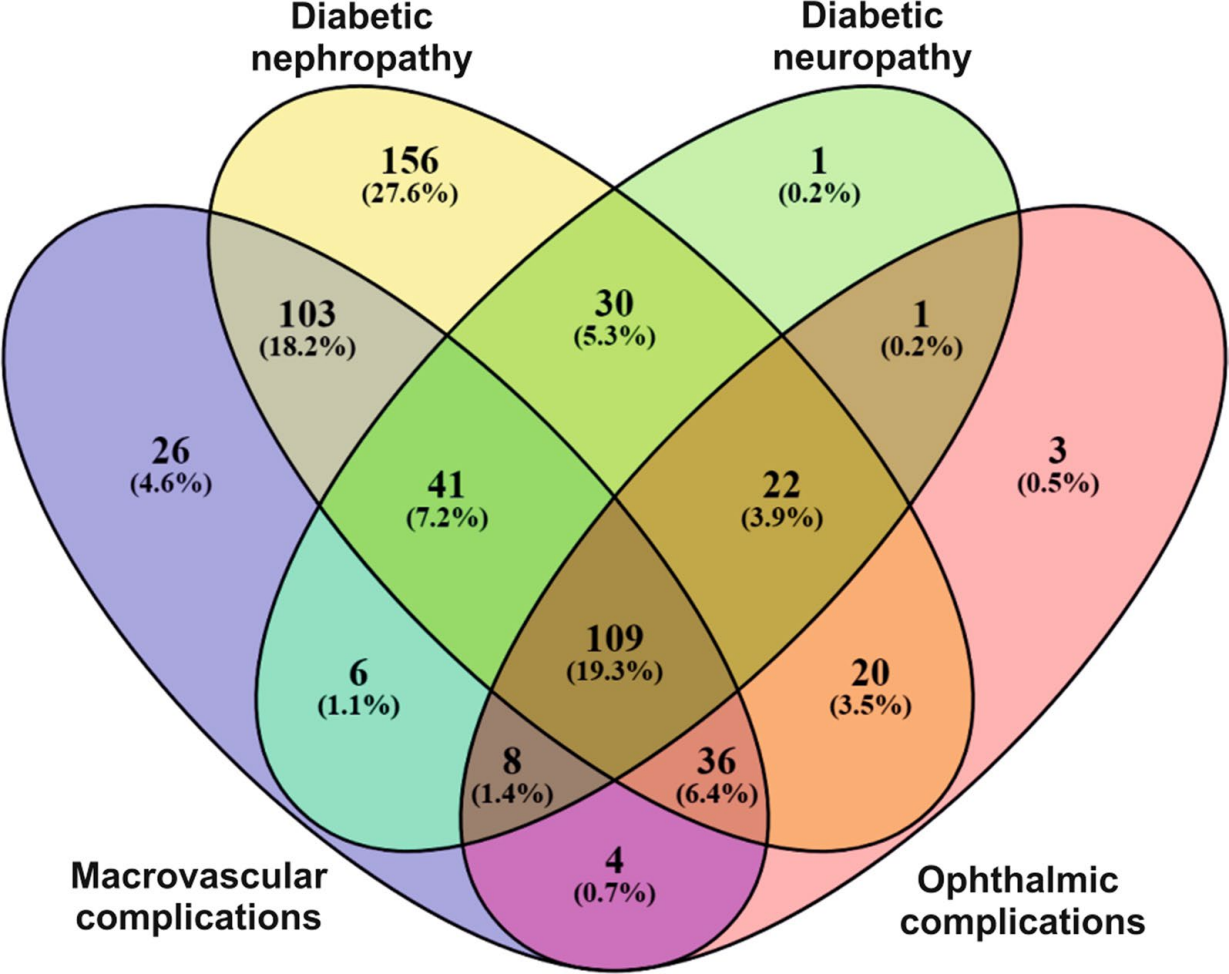
Venn diagram showing the distribution and overlap of cases among the analyzed T2DM complication groups

We studied a cohort of 601 T2DM patients of which 241 were men and 360 women aged 22 to 82 years (average age 56.86 ± 10.24 years) stratified as controls or cases in four complication groups tested (diabetic nephropathy n = 601, diabetic neuropathy n = 600, ophthalmic complications n = 601 and macrovascular complications n = 559), based on corresponding diagnosis or medical events experienced after the onset of T2DM. A phenotype-based distribution of subjects among different complication groups is shown in Fig. 1. After inspection of patient anthropometric data, we observed that T2DM patients experiencing ophthalmic complications and diabetic neuropathy were significantly younger compared to the group of patients without particular complications while those with diabetic nephropathy, macrovascular and ophthalmic complications were characterized by longer duration of diabetes. As expected, median glycated hemoglobin levels were higher in cases (patients with specific complications) compared to controls (patients without the same diabetes complication) except for the diabetic nephropathy group (Table 1).

**Table 1:** Characteristics of the study participants

	Neuropathies			Nephropathies			Ophthalmic complications			Macrovascular complications		
	Cases (n = 218)	Controls (n = 382)	*P* value	Cases (n = 517)	Controls (n = 84)	*P* value	Cases (n = 203)	Controls (n = 398)	*P* value	Cases (n = 333)	Controls (n = 226)	*P*-value
Mean age, years ± SD	55.28 ± 10.24	57.72 ± 10.13	4.61E−03	56.59 ± 10.25	58.57 ± 10.02	1.02E−01	54.38 ± 10.65	58.10 ± 9.80	6.59E−05	56.36 ± 9.88	56.52 ± 10.63	7.58E−01
Female/male, n (%)	120/98 (55.05/44.95)	239/143 (62.57/37.43)	7.85E−02	312/205 (60.35/39.65)	47/37 (55.92/44.05)	5.08E−01	114/89 (56.16/43.84)	245/153 (61.56/38.44)	2.21E−01	190/142 (57.23/42.77)	149/77(65.93/34.07)	4.80E−02
Mean BMI, kg/m^2^ ± SD	33.27 ± 6.29	33.09 ± 5.96	7.39E−01	33.37 ± 6.00	32.21 ± 6.50	5.18E−02	33.23 ± 6.22	33.2 ± 6.02	8.11E−01	33.46 ± 6.13	32.79 ± 5.97	2.41E−01
Median HbA1c, % (IQR)	7.20 (1.70)	6.90 (1.39)	3.93E−05	7.10 (1.54)	7.00 (1.83)	4.15E−01	7.30 (1.95)	6.91 (1.35)	1.55E−05	7.10(1.85)	6.90(1.15)	3.08E−05
Diabetes duration, years ± SD	6.72 ± 6.09	6.30 ± 4.50	4.07E−01	7.05 ± 6.32	4.79 ± 5.31	3.29E−05	5.95 ± 5.53	6.29 ± 4.45	1.21E−02	5.40 ± 6.09	5.74 ± 4.97	5.88E-03

*SD* standard deviation, *BMI* body mass index, *HbA1c* hemoglobin A1C, *IQR* interquartile range

**Fig. 2:**
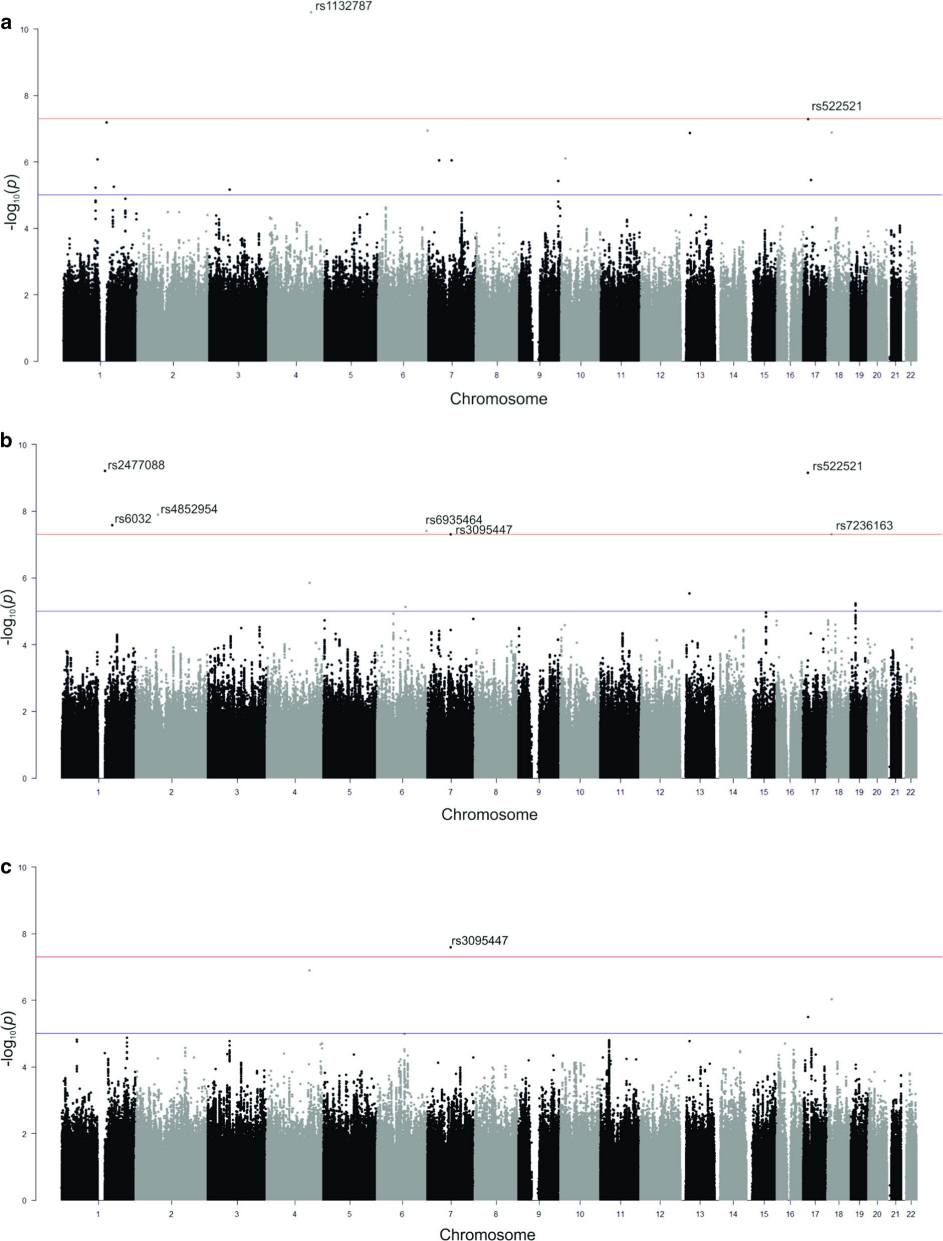
Manhattan plots for GWAS of T2DM complications. **a** Diabetic neuropathies, **b** macrovascular complications, **c** ophthalmic complications. X-axis shows chromosomal positions. Y-axis shows –log10 *P*-values. The red lines indicate a genome-wide significant threshold of *P* < 5 × 10^−8^, and the blue lines denote a suggestive significance threshold of *P* < 5 × 10^−5^. Association signals that reached genome-wide significance are denoted by reference SNP ID number

After quality control and filtering 5 378 539 SNPs were used for further testing in each T2DM complication group. The total genotyping rate was > 0.99 in all of the tested T2DM complication groups. The genomic inflation factor was negligible in all data sets based on median chi-squared statistics: 1.02 for neuropathies, 1.02 for macrovascular complications, 1.01 for ophthalmic complications, and 1.00 for nephropathies. After adjustments for age, sex, BMI, diabetes duration, median HbA1c, and medications used ten susceptibility loci were identified for different T2DM complications (Fig. 2), among them rs1132787 (*GYPA*) and rs522521 (*LOC105371557)* showed an association with diabetic neuropathy, rs2477088 (*PDE4DIP),* rs522521 (*LOC105371557),* rs4852954 (*NAT8)*, rs6032 (*F5*), rs6935464 (*RPS6KA2*), rs7236163 (*ZNF519),* rs3095447 (*CCDC146*) were significantly associated with macrovascular complications, and only variant rs3095447 (*CCDC146*) was related to a greater risk of ophthalmic complications, while no significant hits were found for diabetic nephropathy (Table 2, Additional file 2).

**Table 2:** Susceptibility loci associated with type 2 diabetes mellitus complications

CHR	Closest gene	SNP	A1/A2	OR (95% CI)	*P*-value	MAF cases/controls	MAF European
*Diabetic neuropathy*							
4:145030546	*GYPA*	rs1132787	T/C	2.71 (2.02–3.64)	3.23E−11	0.37/0.16	0.31
17:15733545	*LOC105371557*	rs522521	A/C	0.49 (0.38–0.64)	5.07E−08	0.37/0.56	0.70
*Macrovascular complications*							
1:144936353	*PDE4DIP*	rs2477088	T/C	2.50 (1.87–3.34)	6.11E−10	0.51/0.32	0.58
17:15733545	*LOC105371557*	rs522521	A/C	0.42 (17.32–0.56)	6.95E−10	0.40/0.60	0.70
2:73870010	*NAT8*	rs4852954	T/C	2.27 (2.71–3.01)	1.26E−08	0.50/0.31	0.61
1:169511555	*F5*	rs6032	T/C	2.12 (1.63–2.77)	2.62E−08	0.56/0.37	0.73
6:167114208	*RPS6KA2*	rs6935464	A/G	2.25 (6.69–3.01)	3.89E−08	0.44/0.26	0.57
18:14150724	*ZNF519*	rs7236163	T/C	2.14 (18.63–2.82)	4.97E−08	0.46/0.27	0.58
7:76764970	*CCDC146*	rs3095447	A/C	2.16 (7.64–2.84)	4.98E−08	0.55/0.37	0.79
*Ophthalmic complications*							
7:76764970	*CCDC146*	rs3095447	A/C	2.18 (1.66–2.87)	2.55E−08	0.61/0.40	0.79

*CHR* chromosome and base pair position in Human Genome build hg19, *A1* minor allele, *A2* major allele, *OR* odds ratio for the minor allele, *CI* confidence interval 95%, *MAF* minor allele frequency, *MAF* *European* minor allele frequency observed in the European population [31]

The functional consequences of the identified variants were evaluated by the eQTL analysis, which was conducted in GTEx database by focusing only on those tissue types that may be relevant or even damaged according to the etiology of the disease [29]. Three out of eight novel allelic variants identified in this study appeared to be significantly associated with the expression of multiple nearby genes: rs4852954 (*ALMS1*, *DUSP11*, *NAT8, ALMS1P1, TPRKB, ALMS1-IT1, RP11-434P11.2),* rs7236163 (*ZNF519, RP11-411B10.2*), and rs3095447 (*RP11-467H10.1, FGL2, PMS2P9, GSAP, SPDYE18, FAM185BP, CCDC146, UPK3BP1*). See Additional file 3 for the full list of the identified associations.

### Targeted analysis

In order to investigate if previously reported associations in other populations are also true in our study cohorts, we performed a targeted analysis in the same subjects and stratification in complication groups (diabetic neuropathy, macrovascular complications, ophthalmic complications, and diabetic nephropathy) (Table 1) by performing an association analysis for 194 candidate variants in total. Information on previously known allele-trait associations reported in both GWAS Catalog and GWAS Central was used for the selection of risk variants associated with at least one group of the tested T2DM complications (see Additional file 1 for a full list of selected candidate SNPs). The genotyping rate in all of the complication groups analyzed was > 0.99. By applying the targeted approach we managed to replicate two significant associations for diabetic neuropathy rs3761980 and rs80028505, both mapping to *MAPK14* loci and one significant hit (rs136161*, APOL1*) for diabetic nephropathy (Table 3) in our study cohort.

**Table 3:** Candidate variants showing a significant association with type 2 diabetes complications

CHR	Closest gene	SNP	A1/A2	MAF cases/controls	MAF European	OR (95% CI)	*P*-value	FDR	References
*Neuropathies*									
6:35993906	*MAPK14*,* SLC26A8*	rs3761980	G/A	0.13/0.09	0.10	1.58 (1.08–2.33)	1.94E-02	3.88E-02	Meng et al. [49]
6:35998388	*MAPK14*	rs80028505	T/C	0.13/0.09	0.11	1.58 (1.08–2.33)	1.94E-02	3.88E-02	Meng et al. [49]
*Nephropathies*									
22:36657432	*APOL1*	rs136161	G/C	0.47/ 0.29	0.40	2.00 (1.40–2.86)	1.41E-04	6.93E-03	Iyengar et al. [50]

*CHR* chromosome and base pair position in Human Genome build hg19, *A1* minor allele, *A2* major allele, *OR* odds ratio for the minor allele, *CI* confidence interval 95%, *FDR* false discovery rate by Benjamini & Hochberg method, *MAF* minor allele frequency, *MAF* European: minor allele frequency observed in the European population [31]

## Discussion

Here we present the results of the genome-wide association study for T2DM complications performed in a population of Latvia for the first time, revealing 10 susceptibility loci for T2DM complications, including diabetic neuropathy, macrovascular and ophthalmic complications. As in other reports aimed to identify the risk factors of T2DM complications [15, 32], the control group of our study consisted of T2DM patients with no evidence of the complication type of interest instead of conventional healthy subjects, since the implementation of healthy controls would rather reveal genetic associations with the diagnosis of T2DM itself, not the T2DM complications.

We found two novel variants (rs1132787 and rs522521) associated with diabetic neuropathy and none of them have been linked to any disease or specific phenotype before. Variant rs1132787 is located within the 3′ UTR of a gene coding for glycophorin A coding (*GYPA*). Glycophorin A is the major erythrocyte membrane sialoglycoprotein. Although it has not been directly associated with susceptibility to any T2DM-related condition before, studies report a significant upregulation of the *GYPA* gene in the dorsal root ganglia of a mouse model of T2DM and the metabolic syndrome, and even type 1 diabetes with diabetic polyneuropathy [33, 34]. Moreover, copy number variation overlapping *GYPA* has been already linked to body mass index, obesity, and obesity-related traits, such as weight, hip circumference, and waist circumference, providing more evidence for the potential contribution of *GYPA* in the development of diabetic complications [35]. Because of the multiple evidence of the implication of *GYPA* gene in neuropathies and metabolic traits, and the potential functional consequence of the top variant rs1132787 located within the 3′UTR of the gene, we consider the *GYPA* gene as the first candidate for future functional validation studies. Nevertheless, credible evidence for this association should be established first by performing a replication study with larger sample size. The other identified risk variant rs522521 is located near the poorly characterized gene *LOC105371557* with yet unknown function.

In total, seven variants reached genome-wide significance for the association with macrovascular complications of T2DM. The strongest association was exhibited by an intron variant of the Phosphodiesterase 4D Interacting Protein coding gene (*PDE4DIP*). Although the rs2477088 variant has not been previously linked to any T2DM manifestations, *PDE4DIP*, also known as myomegalin or cardiomyopathy-associated protein 2, is a well-known contributor of the microtubule control process [36] and some previous evidence exist indicating on the potential role of the gene in macrovascular diseases and T2DM. The exome sequencing has revealed a rare variant of *PDE4DIP*, which significantly increases the risk of ischemic stroke [37], moreover, CpG island methylation in leukocytes annotated to *PDE4DIP* contributes to the epigenetic fingerprint of myocardial infarction [38], and finally, the gene is also significantly downregulated in liver of T2DM patients [39]. Another risk allele (rs4852954) identified in the analysis of macrovascular complications is located near the N-Acetyltransferase 8 coding gene (*NAT8*) and has been previously associated with systolic blood pressure and renal function in the Estonian population [40] which is genetically close to the Latvian population [41]. Although *NAT8* gene has not been linked with T2DM macrovascular complications before, it is considered to be a susceptibility locus for diabetic kidney disease [42]. In addition, we found the coagulation factor 5 coding gene (*F5*) among the risk loci of macrovascular complications. The variant rs6032 is located only around 7 kb from the Factor V Leiden (rs6025), which has been strongly associated with ischemic stroke and incident venous thrombosis before [43, 44], therefore we may speculate that in rs6032 carriers manifestation of the trait are amplified by the presence of T2DM. Although rs6032 is the only missense variant among the top hits, it is categorized as benign according to SIFT and PolyPhen. Nevertheless, the variant is in the LD with rs4524, the risk variant for venous thromboembolism (OR = 1.14; CI = 1.11–1.16) [45].

Finally, 4 more loci showed genome-wide significance for the association with macrovascular complications, among them rs6935464, located within the *RPS6KA2* gene coding for Ribosomal Protein S6 Kinase A2, which is involved in cardiac myocyte stress responses and even considered as a therapeutic target for the prevention of heart failure [46], and rs3095447, an intron variant of Coiled-Coil Domain Containing 146 gene (*CCDC146*) which is also the only significant hit for ophthalmic complications in our data. The last two variants (rs522521 and rs7236163) identified in the analysis of macrovascular complications are located in the intergenic regions near genes *LOC105371557* and *ZNF519* respectively, and both have not been linked to any disease before.

We noticed two of the variants (rs522521, rs3095447) appearing among the top hits of multiple complication groups, which seems rational since a number of patients had experienced more than one type of complication, resulting in a notable overlap of patients among four tested phenotype groups (Fig. 1). This finding may be also explained by similar etiologic characteristics between microvascular diabetic complications involving small vessels (neuropathy, nephropathy, ophthalmic complications) and macrovascular complications related to large vessel damage, with chronic hyperglycemia as the main cause of all these comorbidities. Moreover, microvascular and macrovascular complications tend to be strongly interconnected, and the damage of small vessels may contribute to the manifestations of heart disease in diabetes [7], which coincides with our data showing only a small number of T2DM patients corresponding to one complication group only, while 18% (n = 109) of T2DM patients included in this study had experienced multiple manifestations during the observation period, corresponding to all four analyzed complication types.

According to Ensembl Variant Effect Predictor, most of the identified variants are intronic, though rs1132787, which is located in the enhancer site, and rs4852954 laying within the promoter flanking region may disrupt the functions of regulatory elements and therefore modulate the gene expression patterns. Some of the genes affected by the identified eQTLs (Additional file 3) are previously associated with the performance of the vascular system, for instance, expression of Fibrinogen-like protein 2 coding gene (*FGL2*) in endothelial cells has been previously linked to microthrombosis and cardiac impairment in rats with T2DM [47], and Alstrom Syndrome Protein 1 coding gene (*ALMS1*) is associated with Alström syndrome and characteristic dilated cardiomyopathy [48]. According to GTEx data, rs3095447 negatively correlates with *FGL2* expression in heart and artery, while rs4852954 is linked to lower *ALMS1* levels in whole blood and skin tissue, suggesting the possible functional implication of these variants in the pathogenesis of the disease. In order to explore additional effects of the top hits, quantitative phenotype analyses were performed, revealing significant associations between rs6935464, rs6032, rs3095447, rs4852954, rs7236163, rs522521, and median HbA1c levels. These data suggest that the identified genetic loci may serve as markers for both, development of T2DM complications and alterations in HbA1c levels, though the particular analysis should be repeated in a different cohort where the variable impact of different anti-diabetic medications should also be considered.

By performing targeted analysis of candidate variants we managed to replicate three associations with T2DM complications in the population of Latvia. Both variants (rs3761980 and rs80028505) showing an association with T2DM neuropathy are located near the Mitogen-activated protein kinase 14 coding gene (*MAPK14*) and have been previously linked to increased risk of diabetic foot ulcers in the report of The Genetics of Diabetes Audit and Research in Tayside Scotland (GoDARTS) project [49]. In addition, we found a significant association of rs136161 located in the Apolipoprotein L1 coding gene (*APOL1*) with T2DM nephropathy, which has been already linked to an advanced diabetic kidney disease across multiple ethnic groups [50]. Although we have observed smaller effect sizes for rs3761980 and rs80028505 comparing to other GWAS and much larger effect for rs136161, the previously reported odds ratio values for all three variants fit within the 95% confidence intervals calculated in our study [49, 50].

This study has several limitations, though the small sample size is the primary limiting factor in the risk variant discovery, which may result in an insufficient statistical power for the detection of rare variants with small effect sizes. This may also explain why variants with large effect sizes (OR up to 2.71) are markedly represented among the significant results of our study. Although the retrospective distribution of cases and controls in the analysis of T2DM neuropathy and ophthalmic complications was relatively balanced, the lack of significant hits in the analysis of diabetic nephropathy may be explained by the high incidence of the specific type of complication among the study participants (86%), leaving the size of the control group too small for the identification of true associations. Additionally, the suboptimal case-to-control ratio in the analysis of macrovascular complications may affect the statistical power of the study and explain the relatively high number of associations identified in the specific complication group. Due to the limited number of study subjects, the follow-up period was not fixed or set as inclusion criteria, though it was fitted as a covariate in the association analysis. Since the duration of diabetes is one of the strongest risk factors for the development of vascular complications [51], the use of an equal observation period in all cases would reduce the residual variability and improve the quality of this study.

Successful integration of genotyping data with longitudinal phenotypic information produced from several national health registries has provided strong support for 10 loci showing a genome-wide significance for the association with T2DM complications, some of them with already known importance to the comorbid conditions analyzed. We believe that these findings provide deeper insight into the pathogenesis of T2DM complications and suggest novel candidate genes for further functional studies, while our targeted approach highlights several susceptibility loci showing a directionally consistent impact on phenotype in multiple populations.

## Conclusions

Using the genome-wide genotyping approach this study identified ten novel associations with T2DM complications, including *GYPA* (rs1132787) in diabetic neuropathy, *PDE4DIP* (rs2477088), *NAT8* (rs4852954), *F5* (rs6032), *RPS6KA2* (rs6935464) in macrovascular complications, and *CCDC146* (rs3095447) in ophthalmic complications. Meanwhile, the candidate gene analysis demonstrated a strong association for diabetic neuropathy (*MAPK14:* rs3761980, rs80028505), and diabetic nephropathy (*APOL1:* rs136161), proving the contribution of these risk loci in the pathogenesis of diabetic complications across various populations.

## Supplementary information

**Additional file 1:** List of all candidate SNPs selected for targeted association analysis.

**Additional file 2:** Quantile-quantile plots for GWAS of T2DM complications.

**Additional file 3:** List of significant associations obtained in eQTL analysis.

## Data Availability

The majority of the data generated and analysed during this study are included in this published article and its supplementary information files. The raw genotyping data are under restricted access from LGDB, nevertheless, they are available for research purposes from the corresponding author on reasonable request.

## Abbreviations

BMI: Body mass index
CI: Confidence interval
eQTL: Expression quantitative trait locus
GWAS: Genome-wide association study
HbA1c: Hemoglobin A1C
HR: Hazard ratio
ICD-10: 10Th revision of the International Statistical Classification of Diseases and Related Health Problems.
LGDB: Genome Database of the Latvian Population
MAF: Minor allele frequency
OR: Odds ratio
SD: Standard deviation
SNP: Single nucleotide polymorphism
T2DM: Type 2 diabetes mellitus
UTR: Untranslated region
