# Supplementary material for: Novel susceptibility loci identified in a genome-wide association study of type 2 diabetes complications in population of Latvia
Source: BMC Med Genomics. 2021 Jan 11;14:18. doi: 10.1186/s12920-020-00860-4 (PMC7802349; doi:10.1186/s12920-020-00860-4)
Supplement: Supplementary file 2 — Additional file 2: Quantile-quantile plots for GWAS of T2DM complications. [file 12920_2020_860_MOESM2_ESM.pdf]

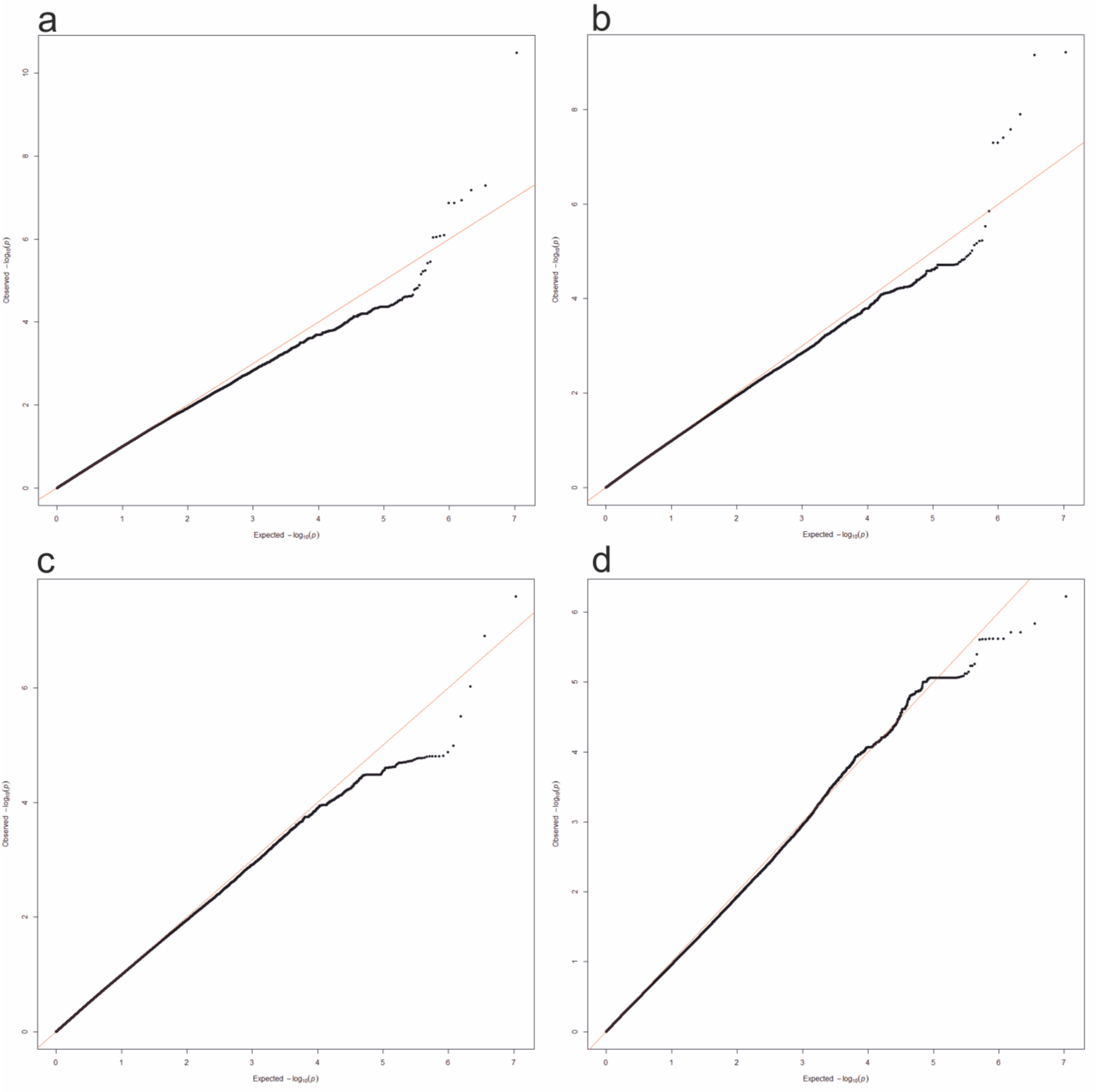

Additional file 2. Quantile-quantile plots for GWAS of T2DM complications. QQ plots showing the distribution of observed (black dots) and expected (red line) p-values in GWAS results for (a) diabetic neuropathy, (c) macrovascular complications, (c) ophthalmic complications and (d) diabetic nephropathy. The plots show the negative of the base 10 logarithms of expected p-values on the x-axis and negative of the base 10 logarithms of observed p-values on the y-axis.
